# Vitamin C Protects Chondrocytes against Monosodium Iodoacetate-Induced Osteoarthritis by Multiple Pathways

**DOI:** 10.3390/ijms18010038

**Published:** 2016-12-27

**Authors:** Pu-Rong Chiu, Yu-Chen Hu, Tzu-Ching Huang, Bau-Shan Hsieh, Jou-Pei Yeh, Hsiao-Ling Cheng, Li-Wen Huang, Kee-Lung Chang

**Affiliations:** 1 Graduate Institute of Medicine, College of Medicine, Kaohsiung Medical University, Kaohsiung 80708, Taiwan; cprong@gmail.com (P.-R.C.); huangtavia@gmail.com (T.-C.H.); 2 Department of Biochemistry, School of Medicine, College of Medicine, Kaohsiung Medical University, Kaohsiung 80708, Taiwan; chingshouhu@gmail.com (Y.-C.H.); hsiehbs@gmail.com (B.-S.H.); masakykaou@hotmail.com (J.-P.Y.); 3 Division of Pulmonary and Critical Care Medicine, Department of Internal Medicine, Kaohsiung Medical University Hospital, Kaohsiung Medical University, Kaohsiung 80756, Taiwan; Chenghl.tanya@gmail.com; 4 Department of Medical Laboratory Science and Biotechnology, Kaohsiung Medical University, Kaohsiung 80708, Taiwan; 5 Institute of Medical Science and Technology, College of Sciences, National Sun Yat-sen University, Kaohsiung 80424, Taiwan; 6 Department of Medical Research, Kaohsiung Medical University Hospital, Kaohsiung Medical University, Kaohsiung 80756, Taiwan

**Keywords:** chondrocyte, interleukin, matrix metalloproteinase, osteoarthritis, vitamin C

## Abstract

Osteoarthritis (OA) is the most prevalent joint disease. Dietary intake of vitamin C relates to a reduction in cartilage loss and OA. This study examined the efficacy of vitamin C to prevent OA with the in vitro chondrosarcoma cell line (SW1353) and the in vivo monosodium iodoacetate (MIA)-induced OA rat. Results demonstrated that, in SW1353 cells, treatment with 5 μM MIA inhibited cell growth and increased oxidative stress, apoptosis, and proteoglycan loss. In addition, the expression levels of the pro-inflammatory cytokines IL-6, IL-17A, and TNF-α and matrix metalloproteinases (MMPs) MMP-1, MMP-3, and MMP-13 were increased. All of these MIA-induced changes could be prevented with treatment of 100 μM vitamin C. In an animal model, intra-articular injection of MIA-induced cartilage degradation resembled the pathological changes of OA, and treatment of vitamin C could lessen these changes. Unexpectedly, vitamin C’s effects did not strengthen with the increasing dosage, while the 100 mg/kg dosage was more efficient than the 200 or 300 mg/kg dosages. Vitamin C possessed multiple capacities for prevention of OA progress, including a decrease in apoptosis and in the expression of pro-inflammatory cytokines and MMPs in addition to the well-known antioxidation.

## 1. Introduction

Osteoarthritis (OA) is one of the most common joint diseases in the world and is a major cause of disability in the aging population [1]. It is a type of joint disease resulting from the breakdown of components of joint cartilage, including chondrocytes, aggrecan, and type II collagens. Chondrocyte impairment will break the balance between the anabolism and catabolism of extracellular matrix; this process plays a critical role in the progression of OA [2]. Evidence from in vivo and in vitro studies indicates that the damaged chondrocytes can produce and/or respond to a number of free radicals, cytokines, and chemokines [3]. Elevated levels of pro-inflammatory cytokines, such as interleukin-1β (IL-1β), IL-6, IL-15, IL-17, and tumor necrosis factor-alpha (TNF-α), have been reported in human OA patients [4]. These elevated cytokines were proposed to induce cytokine expression sequentially and then activate chondrocytes to synthesize matrix metalloproteinase (MMPs) and aggrecanases [5], which lead to increased cartilage degradation [1]. It also reported that chondrocyte apoptosis related to OA was due to hypocellularity in articular cartilage [6], which could be induced by free radicals or pro-inflammatory cytokines through Bax up-regulation, cytochrome *c* release, or activation of caspase-9 and caspase-3 [7,8].

Vitamin C (Vit. C) is a water-soluble vitamin with highly effective antioxidant properties due to reactivity with numerous aqueous free radicals and reactive oxygen species (ROS) [9,10]. Studies indicated that dietary intake of vitamin C was associated with a reduction in the risk of cartilage loss and OA in humans, which was related to its capacity against oxidative stress [11,12,13]. However, it is still unknown whether vitamin C has additional effects on the prevention of OA progression.

The aim of the present study was to examine the efficacy of vitamin C to prevent OA, as well as to address the effects on the production of inflammatory cytokines and degradation enzymes and apoptosis and to investigate the antioxidant properties. The human chondrosarcoma cell line (SW1353) has been used for the study of OA worldwide and its MMPs can be activated by lower levels of IL-1β like human primary chondrocytes [14]. Monosodium iodoacetate (MIA), an inhibitor of glyceraldehyde-3-phosphate dehydrogenase, can cause chondrocyte death [15]. It was recognized as a good model, by the intra-articular injection of MIA into the articular cartilage of rodents, for the study of human OA, as the loss of articular cartilage in rodents is similar to that noted in human OA. Therefore, SW1353 cell line culture and MIA-induced OA of rat models were used for in vitro and in vivo experiments, respectively, of this study. Results of this study will provide an assessment of vitamin C application in OA prevention and clues regarding the underlining mechanisms.

## 2. Results

### 2.1. Cell Growth Inhibition

**Figure 1:**
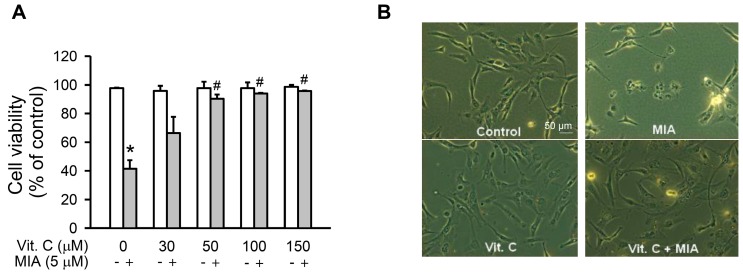
Effect of vitamin C on cell viability in Monosodium iodoacetate (MIA)-treated SW1353 cells. The SW1353 cells were incubated with 30–150 μM vitamin C in the presence or absence of 5 μM MIA for 24 h. (**A**) viable cells were counted by trypan blue exclusion and expressed as a percentage of the control; and (**B**) the morphology of cells with or without 100 μM vitamin C in the presence or absence of 5 μM MIA was observed under a phase-contrast microscopy at 200× magnification. The data are the mean ± S.D. for three separate experiments, each in triplicate. * *p* < 0.05 compared to the untreated control. # *p* < 0.05 compared to the MIA treated group.

To investigate MIA exposure and vitamin C’s protective effects on cell viability of SW1353 cells, the cells were treated with or without 5 μM MIA and/or with different concentrations of vitamin C, as indicated, for 24 h, and then cell viability was assayed. As shown in Figure 1A, cell viability decreased with MIA exposure, while cell viability significantly increased with ≥50 μM vitamin C, even with MIA exposure. However, vitamin C did not increase cell viability in the absence of MIA exposure. Figure 1B shows that the morphology of 5 μM MIA- and 100 μM vitamin C-treated cells was not changed, and it was almost the same as the control, suggesting that vitamin C treatment efficiently protected against MIA-induced cell death.

### 2.2. Oxidative Stress

**Figure 2:**
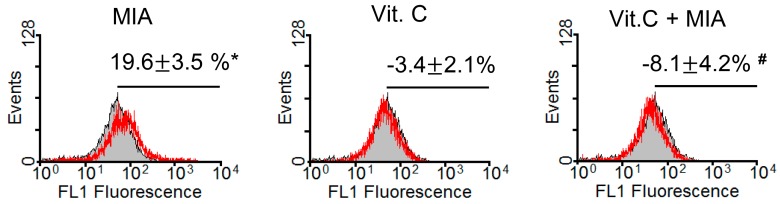
Effects of MIA and/or vitamin C on oxidative stress in SW1353 cells. SW1353 cells were incubated with 100 μM vitamin C in the presence or absence of 5 μM MIA for 8 h, and then production of reactive oxygen species was measured using 2′,7′-dichlorofluorescein diacetate (DCFH-DA) and flow cytometry. The percentages above the panel indicate the percentage of cells showing DCFH-DA fluorescence (reactive oxygen species generation) and are the mean ± S.D. for three separate experiments, each in triplicate. The gray filled area is the untreated control and those delimited by the red lines are the treated groups. * *p* < 0.05 compared to the untreated control group. # *p* < 0.05 compared to the MIA treated group.

Because vitamin C is an antioxidant and could efficiently prevent MIA-induced cell death at concentrations of 100 μM vitamin C as observed above, we examined oxidative stress levels in SW1353 cells after treatment for 8 h with or without 5 μM MIA and/or 100 μM vitamin C. As shown in Figure 2, the oxidative stress levels detected using the fluorescent dye 2′,7′-dichlorofluorescein diacetate (DCFH-DA) were increased by MIA treatment, but this increase was inhibited when vitamin C was added. This indicated that 100 μM of vitamin C could totally block the induction of oxidative stress by 5 μM MIA.

### 2.3. Apoptosis, Cell Cycle Progress, and Apoptosis-Related Proteins

**Figure 3:**
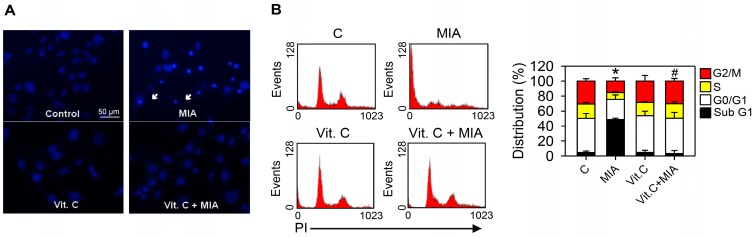
Effects of MIA and/or vitamin C on apoptosis induction and cell cycle progression. SW1353 cells were incubated with 100 μM vitamin C in the presence or absence of 5 μM MIA for 24 h, and then (**A**) apoptosis was determined by Hoechst 33342 staining; and (**B**) the distribution of cells in the different phases of the cell cycle was determined by flow cytometry. In (**A**), the white arrows indicate an apoptotic cell; in (**B**), the down panel indicates the percentage of cell cycle distribution. The results are expressed as the mean ± S.D. for three separate experiments, each in triplicate. * *p* < 0.05 compared to the untreated control. # *p* < 0.05 compared to the MIA treated group.

To determine whether apoptosis was induced by MIA exposure in SW1353 cells and whether vitamin C affected apoptosis, cells were treated with or without 5 μM MIA and/or 100 μM vitamin C for 24 h, and then Hoechst 33342 staining and cell cycle distributions were detected. The Hoechst 33342 showed that exposure for 24 h to MIA induced apoptosis in SW1353 cells, but vitamin C mitigated the effect (Figure 3A). This was confirmed by cell cycle distribution analysis, in which MIA exposure increased the sub-G1 distribution, indicating that apoptosis increased, but addition of vitamin C could inhibit this increase (Figure 3B).

**Figure 4:**
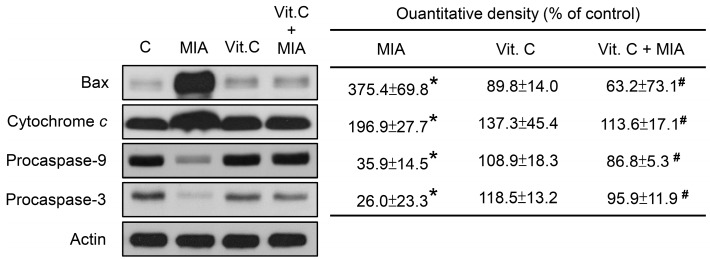
Effects of MIA and/or vitamin C on apoptosis-related protein expression. SW1353 cells were incubated with 100 μM vitamin C in the presence or absence of 5 μM MIA for 24 h, and then the cells were harvested. The proteins were extracted and Bax, cytochrome *c*, procaspase-9, and procasepase-3 were measured. β-actin was used as the internal control. The data of the right panel are expressed as the relative density compared to that in untreated cells (control), which was 100%. The results are the mean ± S.D. for three separate experiments. * *p* < 0.05 compared to the corresponding untreated control. # *p* < 0.05 compared to the corresponding MIA treated group.

To determine whether apoptosis-related proteins were involved in this effect, the cell lysates were subjected to Western blotting. As shown in Figure 4, MIA increased Bax expression and cytochrome *c* release and decreased procaspase-9 and procaspase-3 levels, suggesting that caspase-9 and caspase-3 were activated, which resulted in apoptosis. Consistent with the above observations, vitamin C inhibited the MIA-induced changes.

### 2.4. Proteoglycan Loss

**Figure 5:**
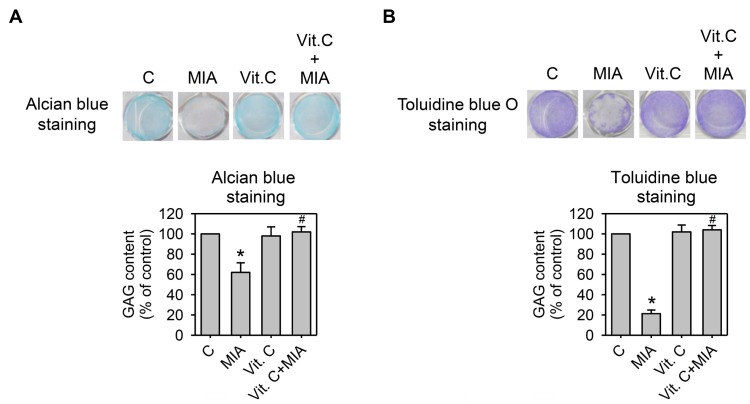
Effect of MIA and/or vitamin C on proteoglycan loss. SW1353 cells cultured in 24-well plates were incubated with 100 μM vitamin C in the presence or absence of 5 μM MIA for 24 h, and then the glycosaminoglycan (GAG) contents in the proteoglycan was determined by (**A**) alcian blue staining; or (**B**) toluidine blue O staining followed by colorimetric assay. The data of each down panel are expressed as the relative density compared to that in untreated cells (control), which was 100%. The results are the mean ± S.D. for three separate experiments. * *p* < 0.05 compared to the untreated control. # *p* < 0.05 compared to the MIA treated group.

Cartilage extracellular matrix is composed primarily of type II collagen and large networks of proteoglycans that contain acidic polysaccharides, such as aggrecan, hyaluronic acid (HA), and chondroitin sulfate [16]. To determine whether proteoglycan was lost or not, SW1353 cells were grown in 24-well plates and treated with or without 5 μM MIA and/or 100 μM vitamin C for 24 h, and then proteoglycans contents were examined by alcian blue or toluidine blue O staining to react with the corresponding acidic polysaccharides. Figure 5A,B show that MIA significantly decreased acidic polysaccharides levels in both staining analyses, and vitamin C significantly inhibited the MIA-induced decrease of acidic polysaccharides. These results indicated that while MIA induced proteoglycan loss, vitamin C inhibited this loss.

### 2.5. Expression of Pro-Inflammation Cytokine and MMP

**Figure 6:**
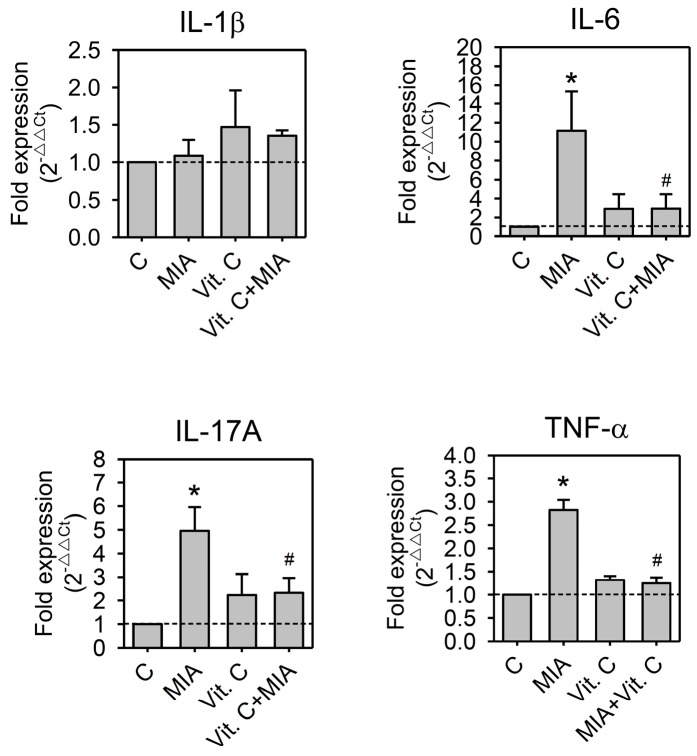
Effects of MIA and/or vitamin C on pro-inflammatory cytokine expressions. SW1353 cells were incubated with 100 μM vitamin C in the presence or absence of 5 μM MIA for 24 h; then, the cells were harvested and the mRNA was extracted, followed by reverse transcription and real-time PCR analysis for IL-1β, IL-6, IL-17A, and TNF-α. The data are expressed as fold changes compared to that in untreated control. The dashed line shows *Y* = 1 of control for reference. The results are the mean ± S.D. for three separate experiments, each in triplicate. * *p* < 0.05 compared to the untreated control. # *p* < 0.05 compared to the MIA treated group.

**Figure 7:**
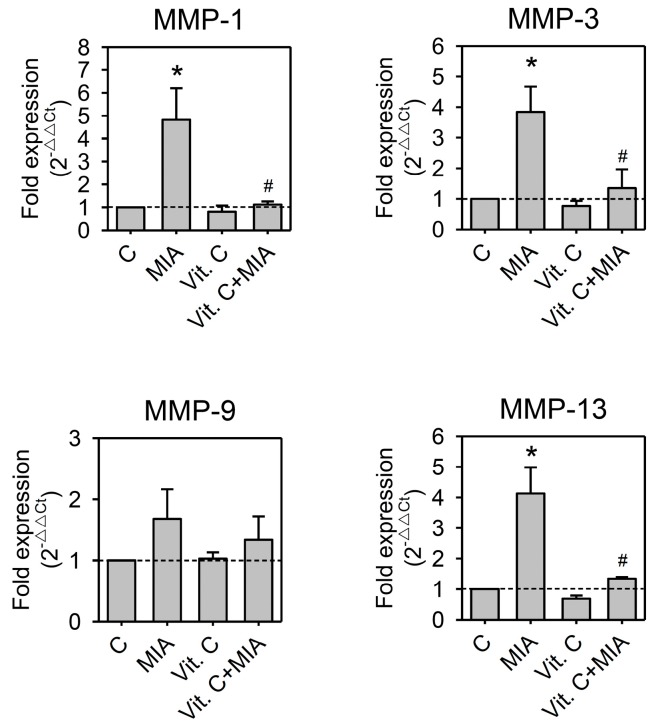
Effects of MIA and/or vitamin C on matrix metalloproteinase (MMP) expressions. SW1353 cells were incubated with 100 μM vitamin C in the presence or absence of 5 μM MIA for 24 h; then, the cells were harvested and the mRNA was extracted, followed by reverse transcription and real-time PCR analysis for MMP-1, MMP-3, MMP-9, and MMP-13. The data are expressed as fold changes compared to untreated control. The dashed line shows *Y* = 1 of control for reference. The results are the mean ± S.D. for three separate experiments, each in triplicate. * *p* < 0.05 compared to the untreated control. # *p* < 0.05 compared to the MIA treated group.

It was reported that inflammatory cytokines participated in the pathogenesis of OA [17,18]. These cytokines induced the synthesis of MMPs, which destroy cartilage components [17,19,20]. To determine whether pro-inflammatory cytokines or MMPs participate in the MIA-induced damages or the protective effects of vitamin C in SW1353, cells were treated with or without 5 μM MIA in the presence or absence of 100 μM vitamin C for 24 h, and then RNA was extracted and followed by reverse transcription and real-time PCR analysis. As shown in Figure 6, MIA increased IL-6, IL-17A, and TNF-α expression but did not change the expression of IL-1β. In addition, MIA also increased MMP-1, MMP-3, and MMP-13 expression but not MMP-9. Similar to these observations above, vitamin C efficiently inhibited MIA-induced changes (Figure 7). These results suggest that inflammatory cytokines and their downstream effectors, MMPs, participate in MIA-induced damages and protection of SW1353 cells by vitamin C.

### 2.6. Articular Cartilage Loss of the MIA-Induced OA in Rats

**Figure 8:**
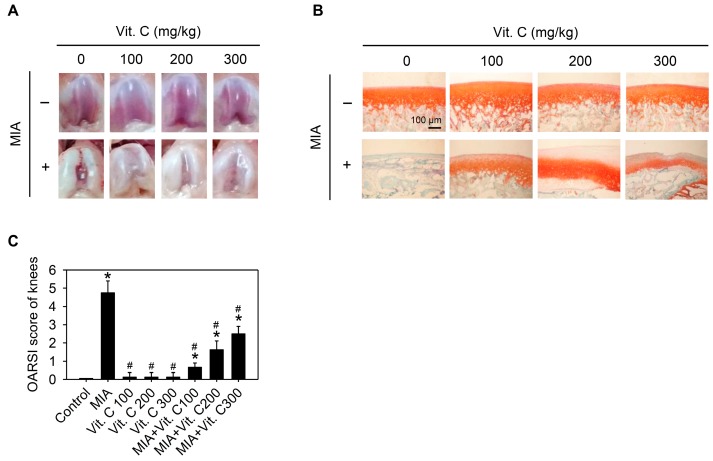
Effect of vitamin C on articular cartilage of rat with MIA-induced OA. The details of MIA-induced OA in rats and subsequent vitamin C treatment are described in the Materials and Methods. After vitamin C treatment for two weeks, the articular cartilage of rats was removed and (**A**) macroscopic observation or (**B**) histologic evaluation or (**C**) Osteoarthritis Research Society International (OARSI) score of each joint occurred, *n* = 10. * *p* < 0.05 compared to the untreated control. # *p* < 0.05 compared to the MIA treated group.

**Figure 9:**
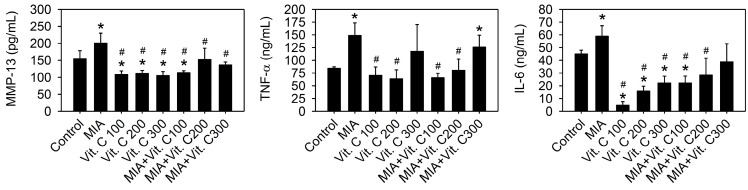
Effect of vitamin C on the serum levels of pro-inflammation cytokines and MMPs in rats with MIA-induced OA. The details of MIA-induced OA in rats and subsequent vitamin C treatment are described in the Materials and Methods. After vitamin C treatment for two weeks, serum levels of IL-6, TNF-α, and MMP-13 were analyzed by enzyme-linked immunoassay *n* = 10. * *p* < 0.05 compared to the untreated control. # *p* < 0.05 compared to the MIA treated group.

Next, we wanted to further confirm that the results found in the cell culture were the same as those in vivo. Therefore, an intra-articular injection of MIA in rats was used as described in the Materials and Methods. Figure 8A shows the morphology of the articular cartilage, in which the MIA-treated group has marked arthritic progression, synovial hypertrophy and cartilage defects, whereas groups with vitamin C intake did not have this defect. The histological results (Figure 8B,C) show that the MIA group had no safranin O staining (red color) and was positive for fast green staining, indicating no acidic proteoglycan cartilage, while the vitamin C-treated groups had smooth joint surfaces with normal articular cartilage and were safranin O positive and with lower Osteoarthritis Research Society International (OARSI) scores, indicating that proteoglycan was not lost. It was noted that vitamin C intake higher than 100 mg/kg per day did not have better effects than 100 mg/kg. Figure 9 shows serum IL-6, TNF-α, and MMP-13 levels were increased by MIA injection and vitamin C decreased the levels regardless of whether MIA was injected or not. These data showed that the observations in the SW1353 cell culture, such as the expression of cytokines and MMPs, were reproducible in the rats of the MIA-induced OA model. Taken together, these results demonstrated that vitamin C could prevent MIA-induced cartilage loss both in vitro and in vivo.

## 3. Discussion

**Figure 10:**
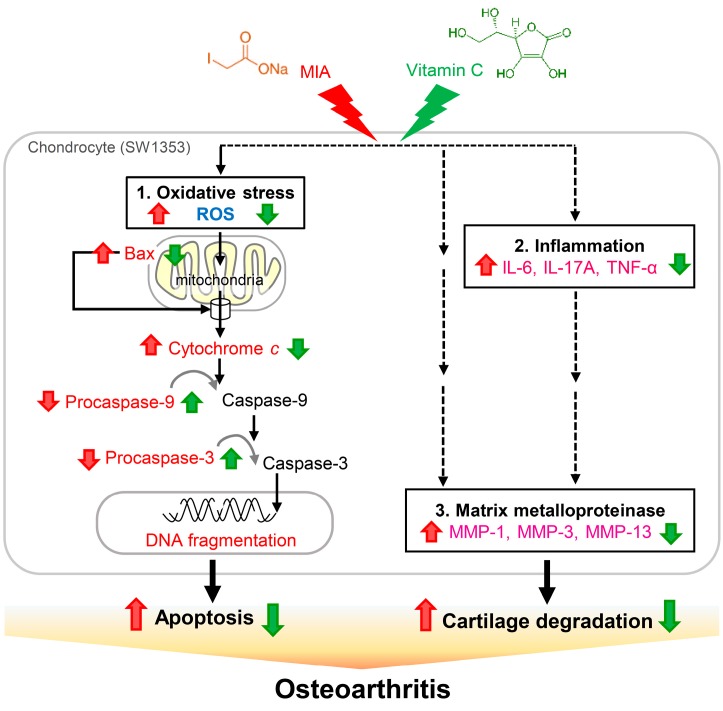
Schematic diagram of vitamin C effects on MIA-treated chondrocytes. The present study demonstrates that, upon 5 μM MIA exposure to SW1353 cells, ROS, Bax, and cytochrome *c* release are increased; however, procaspase-9 and procaspase-3 levels are decreased, indicating that caspase-9 and caspase-3 are activated, which leads to increased apoptosis of chondrocytes (1. Oxidative stress). Additionally, the expressions of pro-inflammation cytokines IL-6, IL-17A, and TNF-α (2. Inflammation), and MMPs MMP-1, MMP-3, and MMP-13 are increased (3. Matrix metalloproteinase), which may cause cartilage degradation. These contribute to the occurrence of osteoarthritis. Interestingly, all of these MIA-induced changes can be decreased with vitamin C treatment. Black solid lines indicated evidences found in this study, dashed lines indicated unknown involved actors. Red 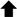: enhanced by MIA; Red 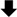: decreased by MIA; Green 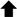: enhanced by Vitamin C; Green 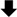: decreased by Vitamin C.

The present study shows that, in SW1353 cells, exposure to 5 μM MIA can inhibit cell growth and increase oxidative stress, apoptosis, and proteoglycan loss. In addition, MIA exposure also significantly increases the expressions of the pro-inflammatory cytokines of IL-6, IL-17A, and TNF-α and the MMPs of MMP-1, MMP-3, and MMP-13. Interestingly, we find that all of these MIA-induced changes can be prevented with treatment of vitamin C at the concentration of 100 μM in SW1353 cells. In an animal model with rats, we find that intra-articular injection of MIA induces cartilage degradation resembling the pathological changes of OA and treatment of vitamin C can lessen these changes (Figure 10). Unexpectedly, the effect of vitamin C is not strengthened with the increasing dosage, while the dosage of 100 mg/kg is more efficient than that of 200 or 300 mg/kg dosages, suggesting that there is an optimal dose of vitamin C for the treatment of OA and that overdose of vitamin C is not beneficial to OA. These findings indicate that, with respect to the inhibition of OA progress, vitamin C possesses multiple benefits, including decrease in apoptosis and in expressions of pro-inflammatory cytokines and MMPs, in addition to the well-known reaction with reactive oxygen species. 

Fibrillation and erosion in cartilage tissue, osteophyte formation at the joint margins, and sclerosis of subchondral tissues are characteristics of the degenerative joint disease, OA [21]. Intra-articular injection of 0.3 or 3 mg MIA to knee joints of Wistar rats induced degenerative lesions with similar histological findings to human OA [22]. Therefore, it was recognized as an appropriate method to mimic lesions of OA in human. Jiang et al. reported that the MIA-induced apoptosis of chondrocytes was mitochondrial-dependent and was by an increase of ROS and activation of caspase [2]. Consistently, our findings of this study also showed that apoptosis was induced by the MIA challenge, which was apparent based on the increase of observed in the Hoechst 33342 staining, the sub G1 cell distribution, cytochrome *c* release, and expression of the apoptotic related protein, Bax, as well as the decrease in procaspase-3 and procaspase-9. Moreover, this study showed vitamin C addition could efficiently inhibit ROS production and apoptosis occurrence and that those results were almost similar to the control group without MIA challenge.

It has been reported that MIA could induce a transient increase in serum IL-1β, IL-6, and IL-10 observed at early time points in mice models [23]. Our present study shows that MIA could enhance the expression of IL-6, IL-17A, and TNF-α in SW1353 cells, which suggests that chondrocytes may contribute to the increase of serum pro-inflammatory cytokines in the MIA-induced OA model of rodent species. Articular cartilage is surrounded with an extensive extracellular matrix, which is mainly composed of proteoglycan (such as aggrecan) and collagen of type II, IX and XI [24]. Chondrocytes are one of the major components of articular cartilage, and they coordinate the anabolism and catabolism of extracellular matrices [25]. Impairment of chondrocytes (apoptosis or necrosis) leads to an unbalance of the extracellular matrix and progression of OA [2]. Several factors are reported to be the main mediators and/or effectors of progressive cartilage loss, including the pro-inflammatory cytokines IL-1, IL-6, IL-17, and TNF, the chemokine IL-8 [18,26], the extracellular matrix degrading enzymes, MMPs, and aggrecanases (a disintegrin and metalloproteinase with thrombospondin motifs, DAMTS), which act as key downstream players in the inflammatory signal cascade [27,28]. Consistent with these reports, our results showed that MIA could significantly increase pro-inflammatory cytokines and MMP expression in chondrocytes, which caused cartilage degradation. An increase in MMPs (particularly MMP-1, MMP-3, and MMP-13) and aggrecanase are reported to augment cartilage matrix leisure [28,29]. In addition, it has been shown that the central role of cytokines, particularly IL-1 and TNF-α, is to cause the destruction of articular cartilage [30]. Mitogen-activated protein kinases (MAPKs) and NF-κB play key roles in the production of these pro-inflammatory cytokines and the downstream signaling events leading to joint inflammation and destruction through the induction of the expression of MMPs and aggrecanases. This study showed that MMP-1, MMP-3, and MMP-13 were remarkably increased, and proteoglycans were lost in MIA-treated chondrocytes. However, vitamin C can block the changes even if their changes are in mass. It needs clarification through further study as to whether the effects of vitamin C are through MAPKs and NF-κB pathways.

The recommended intake doses of vitamin C for males and females is 90 and 75 mg/day, respectively, and the maximum daily intake is recommended as 2000 mg [31]. In humans, plasma vitamin C concentrations after oral intake are tightly regulated. The peak plasma concentration is approximately 200 μM and the steady-state concentration ranges 70–85 μM, even when excessive amounts (3000 mg) of vitamin C are ingested [32,33]. The safe and effective dosage for treating rats is approximately 6.2-fold higher than in humans generally [34]. Accordingly, we used 30–150 μM vitamin C to treat SW1353 cells and 100–300 mg/kg to treat rats. Our results showed that 100 μM of vitamin C could efficiently inhibit all changes of MIA induction in SW1353 cells, and 100 mg/kg of vitamin C is an optimal dose for treatment of MIA-induced OA in rats. Several studies of the past indicated that increased intake of vitamin C could decrease risk of OA progression and cartilage loss in humans, a causal association with its capacity against oxidative stress [11,12,13]. In addition, some studies indicated that enhancement of circulating vitamin C levels was not beneficial to incident radiographic knee OA, and increased its risk [35]. Our study also indicated that higher vitamin C doses (such as 200 or 300 mg/kg) were not better than lower doses (100 mg/kg) in protection against MIA-induced OA of rats. In this study, we demonstrated that vitamin C could inhibit apoptosis, inflammation, and proteoglycans degradation in addition to its well-known capacities of anti-oxidation. However, it is not known why higher doses of vitamin C do not work more efficiently than lower doses do, whether related to anti-inflammation or others. Further experiments are needed to expose the underlying mechanism. 

In summary, the present study demonstrates that MIA exposure induces ROS production, Bax expression and cytochrome *c* release, as well as decreases procaspase-3 and procaspase-9 levels to activate caspase, resulting in apoptosis in SW1353 cells. In addition, expression levels of pro-inflammation cytokines and MMPs are increased. All of those effects can be prevented through pretreatment of vitamin C. The preventive effects of vitamin C in vitro are similar to those observed in vivo of rats with MIA-induced OA models.

## 4. Materials and Methods

### 4.1. Reagents and Antibodies

MIA, Vitamin C (ascorbic acid), other chemicals of analytical grade used, and a protease inhibitor cocktail were purchased from Sigma-Aldrich Co., LLC. (St. Louis, MO, USA). Monoclonal antibody against human cytochrome *c*, procaspase-9, or Bax from mouse, polyclonal antibody against human procaspase-3 from rabbits, polyclonal antibody against human β-actin from goat, and horseradish peroxidase-conjugated antibody were purchased from Santa Cruz Biotechnology Inc. (Santa Cruz, CA, USA).

### 4.2. Cell Culture

The human originating chondrosarcoma cell line, SW1353, was purchased from the Bioresource Collection and Research Center (BCRC) of the Food Industry Research and Development Institute in Hsinchu, Taiwan. Cells were cultured at 37 °C with Dulbecco’s Modified Eagle Medium (DMEM) containing penicillin (100 units/mL), streptomycin (100 μg/mL) (Gibco BRL, Grand Island, NY, USA) and fetal bovine serum (10%) (Hyclone, Auckland, NZ, USA) in 5% CO_2_ incubator. Cells of 10–20 passages were used for experiments and 5 × 10^5^ cells were seeded to 6 cm dishes for 24 h to allow attachment. Then, these attached cells were treated with 30, 50, 100 or 150 μM vitamin C in the presence or absence of 5 μM MIA for a further 24 h, followed by analysis of the influences.

### 4.3. Cell Viability Assay and Morphology

After treatment, cells were harvested and then viable cells were counted using a dye exclusion technique with 0.4% trypan blue (GibcoBRL, Grand Island, NY, USA). Cell morphological changes were observed by 200× magnification under an inverted phase-contrast microscope (Olympus, Tokyo, Japan).

### 4.4. Measurement of Reactive Oxygen Species (ROS)

SW1353 (5 × 10^5^/dish) cells were treated with MIA or MIA combined with vitamin C for 24 h, and then intracellular ROS were detected by using 2′,7′-dichlorofluorescein diacetate (DCFH-DA) (Molecular Probes, Eugene, OR, USA) as described in our previous study [36].

### 4.5. Detection of Apoptosis and Cell Cycle Progress

After treatment, apoptotic cells were stained with Hoechst 33342 and detected at 200× magnification in a Zeiss Axiovert 200 fluorescence microscope (Carl Zeiss Microscopy Ltd., Cambridge, UK) as described in our previous study [36]. The distributions of cells in different stages (Sub-G1, G0/G1, S, and G2/M) of cell cycles were estimated by flow cytometry DNA analysis, as described previously [37].

### 4.6. Western Blot Analysis

Western blots were performed as described in our previous study [36]. Herein, proteins were visualized by chemiluminescence detection (PerkinElmer Life Sciences, Inc., Boston, MA, USA), actin was served as internal control, and data were quantitatively analyzed as compared to that relative in the control (untreated group).

### 4.7. Glycosaminoglycans Staining

Glycosaminoglycans expression was assayed by alcian blue and toluidine blue O staining. Cells were seeded at 5 × 10^4^ per well in a 24-well plate, treated for 24 h with MIA or MIA combined with vitamin C, washed twice with phosphate buffered saline (PBS), fixed with methanol, and then stained for 10 min at room temperature with 1% alcian blue or 0.5% toluidine blue O. Then, the glycosaminoglycan area was measured using a semiautomatic image-analyzing program (Mac Scope, Mitani, Fukui, Japan) or using the alcian blue and toluidine blue O elution. Absorbance of the formazan product was measured at the wavelengths of 595 and 630 nm.

### 4.8. Quantitative Real-Time PCR Analysis

Total RNA was extracted from cells with REzol reagent (Protech, Taipei, Taiwan) according to the manufacturer’s instructions, as described previously [38]. The complementary DNA (cDNA) was synthesized from random primed reverse transcription from 2 μg of total RNA using M-MLV reverse transcriptase (Promega Corporation, Madison, WI, USA) according to the manufacturer’s directions. Real-time PCR, performed on a MiniOpticonTM Real-Time PCR Detection System (Bio-Rad Laboratories, Hercules, CA, USA) using iQTM SYBR^®^ Green Supermix (Bio-Rad Laboratories, Hercules, CA, USA) according to a published procedure [39], was used to confirm results of real-time PCR. mRNA coding for MMP-1, MMP-3, MMP-9, MMP-13, IL-1β, IL-6, IL-17A, and TNF-α were measured by real-time PCR, with β-actin mRNA being amplified as a housekeeping gene. Primer sequences of targets are listed in Table 1. The cycle threshold (*C*_t_) value of the target gene was corrected by the β-actin. Data were calculated and expressed as ΔΔ*C*_t_ [40] by using MJ Opticon Monitor Analysis software version 3.1 (Bio-Rad Laboratories, Hercules, CA, USA).

### 4.9. Animals and Treatments

Male Wistar rats at 4 weeks of age were purchased from BioLASCO Taiwan Co., Ltd. (Charles River Technology, Taipei, Taiwan). Wistar rats at 5 weeks of age (150–170 g) were used. This study was performed in accordance with the Guide for the Care and Use of Laboratory Animals of the United States National Institutes of Health. The protocol for animal use was reviewed and approved by the Institutional Animal Care and Use Committee (IACUC) of Kaohsiung Medical University (Approval No. 97048, 26 August 2008). Eighty male Wistar rats were randomly assigned to eight groups of 10. For induction of OA, rats were anaesthetized by Zoletil 50 (a mixture of Tiletamine and Zolazepam from Virbac, Carros, France) and were then injected into the infra-patella ligament of the left knee with 3 mg MIA solved in 20 μL 0.9% sterile saline. Control animals were given a single intra-articular injection into the left knee of 20 μL 0.9% sterile saline. The MIA rats were randomly assigned to one of four treatment groups, which were untreated or supplemented for 2 weeks with vitamin C. All rats were fed a standard rodent chow (Altromin, Lage, Germany). The vitamin C-treated rats received additional water-dissolved vitamin C as 100, 200, or 300 mg/kg/day (Sigma-Aldrich, St. Louis, MO, USA) by gavage. At the end of the experiments, the rats were sacrificed using CO_2_ and the left leg was removed for histomorphometric analyses. All samples were stored at −80 °C until analyzed.

### 4.10. Histopathology of Joint Tissues: Safranin O and Fast Green Staining

The left leg was removed, embedded in O.C.T. embedding compound, and stored at −80 °C until analyzed. For analysis, 5 μm sections were prepared, fixed in 10% formaldehyde in PBS for 5 min, exposed to 0.001% fast green (FCF) solution for 5 min, and rinsed quickly with 1% acetic acid solution for 10–15 s. Samples were then stained with 0.1% safranin O solution (Sigma-Aldrich, St. Louis, MO, USA) for 5 min and dehydrated and cleared with 95% ethyl alcohol and absolute ethyl alcohol. Images were obtained at 100× magnification using an Olympus microscope (Olympus Corporation, Tokyo, Japan). The histological score of knee joints was assessed by the OARSI cartilage degeneration score [41].

### 4.11. Serum Biomarker Measurements

After rats fainted from CO_2_ gas, blood was collected by heart punchers, and serum was obtained by centrifuging blood samples at 3000× *g* for 15 min, and then divided into aliquots and frozen at −80 °C. There was no repeated freezing and thawing of specimens before measurements. The inflammatory markers of IL-6 and TNF-α were assayed using Rat IL-6 ELISA Kit and Rat-TNFα ELISA Kit (Elisa Kit, Antibody-Sunlong Biotech Co., Ltd., Hangzhou, China), respectively. The ECM degrading enzymes of MMP-13 were assayed using Rat MMP-13 ELISA Kit (Elisa Kit, Antibody-Sunlong Biotech Co., Ltd., Hangzhou, China). All samples were tested in triplicate within each assay.

### 4.12. Statistical Analysis

All statistical analyses were measured by version 6.011 SAS software (SAS Institute Inc., Cary, NC, USA). For comparison of differences between control and treated groups, an X^2^ test was used to analyze cell cycle distribution, and ANOVA followed by Fisher’s Exact Test was used for the others. It was considered as a significant difference when *p*-value is <0.05.

## 5. Conclusions

In conclusion, the present study demonstrates that MIA can induce chondrocyte apoptosis, increase expressions of pro-inflammation cytokines and MMPs, and cause cartilage degradation, which is similar to OA in humans. Vitamin C can prevent against all MIA-induced changes both in vitro and in vivo through anti-apoptosis, anti-inflammation, anti-degradation, and anti-oxidation.

## Figures and Tables

**Table 1:** Primer sets for qPCR analysis.

Primer Name	NCBI Reference Sequence	Primer Sequence (5′→3′)
β-actin	NM_001101.3	F: ATCGGCGGCTCCATCCTG
		R: ACTCGTCATACTCCTGCTTGC
MMP-1	NM_002421.3	F: AGATGTGGAGTGCCTGATGTG
		R: CTTGACCCTCAGAGACCTTGG
MMP-3	NM_002422.3	F: CCACTCTATCACTCACTCACAG
		R: GACAGCATCAAAGGACAAAGC
MMP-9	NM_004994.2	F: CTGGTCCTGGTGCTCCTG
		R: TGCCTGTCGGTGAGATTGG
MMP-13	NM_002427.3	F: GACCCTGGAGCACTCATGTTTC
		R: TCCTCGGAGACTGGTAATGGC
IL-1β	NM_000576.2	F: TGATGGCTTATTACAGTGGCAATG
		R: GTAGTGGTGGTCGGAGATTCG
IL-6	NM_000600.4	F: ACCCCCAATAAATATAGGACTGGA
		R: GAGAAGGCAACTGGACCGAA
TNF-α	NM_000594.3	F: TCAGCAAGGACAGCAGAGGAC
		R: GGAGCCGTGGGTCAGTATGTG
IL-17A	NM_002190.2	F: GGCTGGAGAAGATACTGGTGTC
		R: AGGCTGTCTTTGAAGGATGAGG

MMP: Matrix Metalloproteinase; IL: Interleukin; TNF-α: Tumor Necrosis Factor-alpha; F: Forward primer; R: Reverse primer.

## Abbreviations

OA	Osteoarthritis
MIA	Monosodium Iodoacetate
MMPs	Matrix Metalloproteinases
IL	Interleukin
TNF-α	Tumor Necrosis Factor-alpha
Vit. C	Vitamin C
ROS	Reactive Oxygen Species
ADAMTS	A Disintegrin And Metalloproteinase with Thrombospondin Motifs
MAPKs	Mitogen-Activated Protein Kinases
